# FAT1 weighted MRI: Diffusion meets anatomical imaging and application in thalamic surgery for tremor

**DOI:** 10.1162/imag_a_00139

**Published:** 2024-04-25

**Authors:** Taco Goedemans, Francisca Ferreira, Thomas Wirth, Lonneke van der Weerd, Flavia V. Massey, Marie T. Krüger, Vanessa Milanese, Ashkan Pakzad, Thomas Foltynie, Patricia Limousin, Maarten Bot, Pepijn van den Munckhof, Rick Schuurman, Ludvic Zrinzo, Harith Akram

**Affiliations:** Unit of Functional Neurosurgery, UCL Queen Square Institute of Neurology & The National Hospital for Neurology and Neurosurgery (UCLH), London, United Kingdom; Department of Neurosurgery, Amsterdam UMC, University of Amsterdam, Amsterdam, The Netherlands; Department of Neurology, Strasbourg University Hospital, Strasbourg, France; Department of Neurosurgery, University Medical Center Utrecht, Utrecht University, Utrecht, The Netherlands; Department of Stereotactic and Functional Neurosurgery, University Medical Center, Freiburg, Germany; Department of Neurosurgery, DOMMO Clinic, and Beneficência Portuguesa of São Paulo Hospital, São Paulo, Brazil; Department of Neurosurgery, Mayo Clinic, Jacksonville, FL, United States; Centre for Medical Image Computing, Department of Medical Physics and Biomedical Engineering, University College London, London, United Kingdom

**Keywords:** deep brain stimulation (DBS), diffusion weighted imaging (DWI), tractography, connectivity, fractional anisotropy (FA), movement disorders, radiofrequency Thalamotomy, stereotactic neurosurgery, ventral intermediate nucleus (Vim)

## Abstract

Patient-specific targeting of the Ventral intermediate nucleus (Vim) of the thalamus can be achieved with MR connectivity. Nevertheless, there are several drawbacks to using tractography-based targeting methods to visualise distinct thalamic nuclei (e.g., subjective region of interest selection, and thresholding of resulting tracts and clusters). Fractional anisotropy (FA) mapping, another product of diffusion MRI (dMRI), does not rely on tractography, and could thus be clinically more viable for discerning thalamic anatomy for stereotactic surgery. The aim of this study is to develop and present a hybrid, high-resolution, and high-fidelity imaging modality that combines contrast from FA maps as well as anatomical T1 sequences (FAT1 imaging); and to evaluate FAT1 based Vim-target definition. Imaging and outcome data of 35 consecutive refractory tremor patients who had undergone 43 connectivity guided deep brain stimulation (DBS) and/or radiofrequency thermocoagulation (RF-T) between 2013 and 2021 were included. First, the pre-operatively acquired dMRI and MPRAGE sequences were used to create FAT1 maps in retrospect. Then, an FAT1 based Vim-target was planned by an experienced functional neurosurgeon who was blinded for patient outcome. Finally, to investigate FAT1 based targeting, a post-hoc analysis was carried out of the degree of overlap between the newly created FAT1 based Vim-target, and the volume of tissue activation (VTA, in case of DBS) or lesion volume (in case of RF-T). This degree of overlap was compared between favourable and unfavourable outcome groups: outcomes were measured by experts blinded for imaging data at the last follow-up using a Clinical Global Impression-Improvement score (CGI-I), where a CGI-I score of 1-2 (i.e., FTMTRS improvement of ≥50%) was considered favourable. In 36 of the 43 (84%) performed surgeries (24 DBS and 19 RF-T), FAT1 based Vim-targeting was possible. For the group showing favourable outcome (71% of the patients at a median follow-up of 13 months), the mean amount of overlap between the FAT1 based Vim-target and the VTA or lesion was 42% (±13), versus 17% (±15) for patients with an unfavourable outcome (MD 25%, 95% CI 14–35, p < 0.0001). Retrospective use of FAT1 based Vim-targeting as a tool to predict outcome had a sensitivity of 90%, specificity of 80%, positive predictive value of 90%, and negative predictive value of 80%. In conclusion, FAT1 imaging is a new, high-resolution, and high-fidelity modality that combines diffusion and anatomical MRI. It provides a fast and efficacious way of targeting the ventral intermediate nucleus of the thalamus. In this study, FAT1 based targeting was highly accurate in predicting outcomes after deep brain stimulation and radiofrequency thalamotomy.

## 1 Introduction

The success of stereotactic functional neurosurgery is contingent upon accurate target identification (Horn et al., 2019). This applies to Deep Brain Stimulation (DBS), as well as ablative surgery (lesioning) with Radiofrequency (RF), Gamma Knife (GK), or High-intensity Focused Ultrasound (HiFU) (Dallapiazza et al., 2019).

The Ventral intermediate nucleus (Vim) of the thalamus is an established target for medically refractory tremor (Benabid et al., 1989,^1991^;Dallapiazza et al., 2019;Hariz et al., 2008;Schuurman et al., 2008;Tsuboi et al., 2020). Unlike other commonly used targets in movement disorders (i.e., the Subthalamic Nucleus (STN) and the internal Globus Pallidus (GPi)), the Vim is not readily visualised on conventional MRI (Lemaire et al., 2010). For this reason, targeting the Vim often relies on predefined atlas coordinates and intraoperative testing with macrostimulation during awake surgery to confirm accurate targeting (Schaltenbrand et al., 1977). This approach does not account for inter-individual variability, is uncomfortable for patients, and can be prone to target confirmation errors due to oedema or stun effect during implantation and testing (Ferreira et al., 2021). The ability to directly visualise the Vim can reduce the number of brain passes, improve surgical outcomes, reduce operating time, and allow surgery under general anaesthesia, thus reducing patient discomfort (Ferreira et al., 2021).

Building on previous work (Behrens et al., 2003,^2007^;Pouratian et al., 2011), in 2018 we described a novel method to identify the Vim, based on patterns of cortical and cerebellar connectivity, taking advantage of its central location as a node on the tremor network (connecting the ipsilateral motor cortex with the contralateral cerebellar dentate nucleus). This connectivity-guided approach utilises high angular resolution diffusion imaging (HARDI) and probabilistic tractography (Akram et al., 2018). Some have also adopted this technique (Horn et al., 2019;Muller et al., 2020) while others utilised deterministic algorithms to visualise the dentato thalamic tract, also referred to as the dentato-rubro-thalamic tract (DRT) in surgical targeting (Coenen et al., 2011;Fenoy et al., 2017). Although both approaches have shown promise in visualising distinct thalamic nuclei based on diffusion where anatomical MRI sequences have failed to do so, they require a great deal of local expertise and processing time. Furthermore, these techniques are reliant on accurately defining tractography seeds and regions of interest (ROIs), can result in false positive/negative results (Maier-Hein et al., 2017), can have poor accuracy and reproducibility (in the case of deterministic algorithms (M. V. Petersen et al., 2017)), can be affected by individual anatomical bottlenecks (Ferreira et al., 2021), and arbitrarily set fibre thresholds.

A product of diffusion MRI (dMRI) that does not rely on tractography is fractional anisotropy (FA) mapping. This modality visualises local diffusivity (axonal integrity) reflecting brain microstructure. It has been previously used as a tool in Vim targeting (Lefranc et al., 2015;Sedrak et al., 2011); however, FA maps are often generated from low-quality diffusion tensor imaging data with poor signal-to-noise ratio and significant geometrical distortion. Moreover, accurately fusing these sequences with other imaging modalities (intraoperative stereotactic CT or MRI) to target the Vim poses significant challenges.

**Fig. 1.**
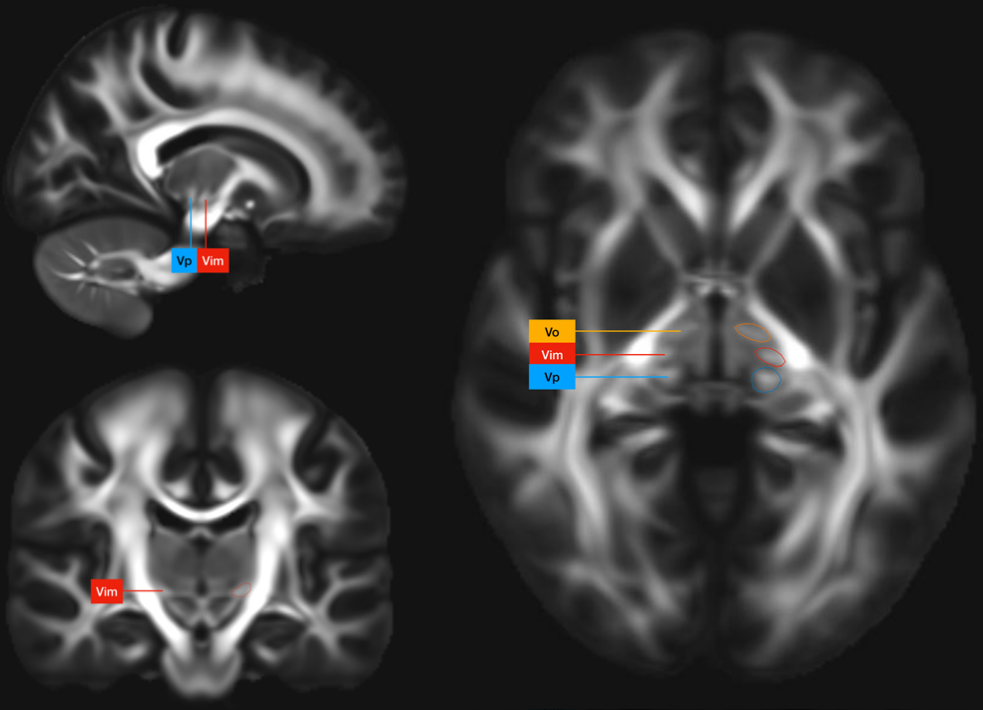
Group average FA map from the HCP 1065 Healthy Subjects Connectome showing contrast between the Ventroposterior sensory thalamic nucleus (Vp), the Ventrointermedial nucleus (Vim), and the Ventrooralis nucleus (Vo).

The development of a hybrid, high-resolution, and high-fidelity imaging modality that combines contrast from FA maps as well as anatomical T1 sequences could provide a clinically viable method for discerning thalamic anatomy for stereotactic surgery. These maps have significantly improved contrast to noise ratio. A clear example of the intrathalamic contrast achieved on high-quality FA maps can be appreciated on the Human Connectome Project 1065 subject (HCP-1065) group average connectome (Fig. 1).

In this work, we set out to 1) develop and present FAT1 imaging as a new imaging modality using patient-specific, preoperative HCP style dMRI and MPRAGE sequences; 2) present the outcomes from 35 consecutive tremor patients treated with connectivity-guided Vim surgery (DBS and RF ablation); and 3) examine FAT1 based targeting compared to the actual target.

## 2 Materials and Methods

### 2.1 Patients

All patients who had undergone connectivity-guided Vim DBS or RF ablation for severe medically refractory Parkinson’s disease tremor (PDT) and Essential Tremor (ET) at the National Hospital for Neurology and Neurosurgery in Queen Square, London from 2013–2021 were included. The cohort included nine previously described patients (Akram et al., 2018). Patient selection was made by a multidisciplinary team of expert movement disorders neurologists and functional neurosurgeons. Formal ethical approval for the current observational study was waived by West London NHS Research Ethics Committee.

### 2.2 Preoperative diffusion weighted and structural MRI acquisition

Due to MRI scanner upgrade, patients included in this study underwent imaging on two scanner systems with two different protocols. The first cohort of patients (2013–2017, n = 9) underwent scanning on a 3T Siemens Magnetom TrioTim Syngo MR B17 with High Angular Resolution Diffusion Imaging (HARDI) with isotropic voxel resolution of 1.5 mm^3^(TR = 12200 ms, TE = 99.6 ms) in a single shell of 128 uniformly distributed diffusion directions with a b-value of 1500 seconds/mm^2^with seven distributed b = 0 acquisitions for registration. A 32-channel head coil was used. To correct for off resonance field distortion, all acquisitions were repeated with a reversed-phase encoding direction (left to right and right to left phase encode) giving a total of 270 volumes acquired. In-plane acceleration was used (GRAPPA factor of 2) with partial Fourier 6/8. Total acquisition time was 62 minutes. This was accompanied by isotropic R1 maps (quantitative 1/T1 maps) generated from multiparameter maps with a resolution of 1 mm^3^(Akram et al., 2018;Harms et al., 2018).

The second cohort of patients (2017–2022, n = 26) underwent imaging on a 3T Siemens Prisma system using standardised Connectomic / HCP style acquisitions as used in the Aging HCP protocol (Bookheimer et al., 2019). These sequences are freely available to download on the HCP website (https://www.humanconnectome.org/storage/app/media/protocols/CCF_Prisma_2016.07.14.zip). They include multi-band, multi-shell HARDI with isotropic voxel resolution of 1.5 mm^3^(multiband factor = 4, TR = 3230 ms, TE = 89.2 ms with two shells of b-values of 1500 and 3000 seconds/mm^2^) in two acquisitions with reverse-phase encode directions in AP and PA. Each shell had 92 diffusion directions with 6 + 7 b0 acquisitions giving a total of 394 volumes acquired. In-plane acceleration was used (GRAPPA factor of 2) with partial Fourier 6/8. Total acquisition time was 22 minutes. This was accompanied by the HCP isotropic MPRAGE volume of 0.8 mm^3^resolution.

These data were preoperatively used for the surgical planning of connectivity-guided thalamic surgery, and postoperatively used to create an FAT1 based Vim-target for the purposes of this study. As described byAkram et al. (2018), the following steps were taken in order:

***High angular resolution diffusion imaging (HARDI) processing***

Image processing was performed as previously described in detail (Akram et al., 2018). In short, the pre-processing steps included the following: first, images were imported from DICOM to NifTI volumes; the susceptibility-induced field distortion was corrected using Topup (FSL, FMRIB’s software library, v5.0) (Andersson et al., 2003;Jenkinson et al., 2012;Smith et al., 2004), after which the output was fed into Eddy (FSL v5.0) for subject movement and Eddy current distortion correction (Andersson & Sotiropoulos, 2016).

***Probabilistic tractography: thalamic segmentation***

To estimate fibre orientation within each voxel, BedpostX (FSL v5.0) was run with up to three crossing fibres estimated ( Hernández et al., 2013 ; Jbabdi et al., 2012 ). Then, registration to the patient’s structural reference image was performed using FLIRT (FMRIB’s Linear Image Registration Tool; 6 degrees of freedom, correlation ratio cost function, and normal search; Jenkinson & Smith, 2001 ; Jenkinson et al., 2002 ). High-performance computing with parallel GPU processing was used for probabilistic tractography ( Akram et al., 2018 ). The following thalamic regions were segmented: the ventrolateral thalamus (VL, i.e., region connected to the ipsilateral primary motor cortex), the ventroposterior thalamus (VP, i.e., region connected to the ipsilateral primary sensory cortex), and the ventral intermediate thalamic nucleus (Vim, i.e., region connected to the contralateral dentate nucleus). To segment these regions, ProbtrackX2 GPU version ( Behrens et al., 2007 ; Hernandez-Fernandez et al., 2016 ) (FSL v.5.0) was run, using the distributions of voxel-wise principal diffusion directions as created in BedpostX. The following previously created (and in patient space transformed) masks were included ( Akram et al., 2018 ):

- Seeds to M1: seed: thalamus; waypoint: primary motor cortex (M1); exclusions: CSF, contralateral cerebrum and ipsilateral cerebellum
- Seeds to S1: seed: thalamus; waypoint: primary sensory cortex (S1); exclusions: CSF, contralateral cerebrum and ipsilateral cerebellum
- Seeds to cerebellum: seed: thalamus; waypoint: contralateral dentate nucleus/cerebellar white matter; exclusions: CSF, contralateral cerebrum and ipsilateral cerebellum

The following settings were applied: sample number = 20000, curvature-threshold = 0.2, step length = 0.5 mm, and subsidiary fibre volume fraction threshold = 0.01. The thalamic seed voxels were then classified according to their connection probability (Behrens et al., 2003,^2007^). Thresholding was applied using*fslmaths*and/or*fsleyes*by the performing neurosurgeon to correct for false positive tracts (Zalesky et al., 2016).

***Microstructural analysis: creating FA maps***

After the diffusion-related pre-processing steps were undertaken, DTIFIT (Diffusion Tensor Imaging local fitting, FSL v5.0) was run (using the b-vectors, b-values, brain masks, and the aligned and undistorted diffusion data), thereby creating high-fidelity, distortion-corrected FA maps.

***Structural T1w MPRAGE imaging***

Structural images were imported from DICOM to NifTI volumes, after which brain extraction was performed using BET (Brain Extraction Tool, FSL v5.0) to further use these images for registration of the diffusion data to structural patient space (Jenkinson et al., 2012;Smith, 2002). For surgical planning, both the structural images and the thalamic segmentation were used to target the Vim (i.e., connectivity-based targeting, see subheading 2.3).

***Creating FAT1 sequences***

**Fig. 2.**
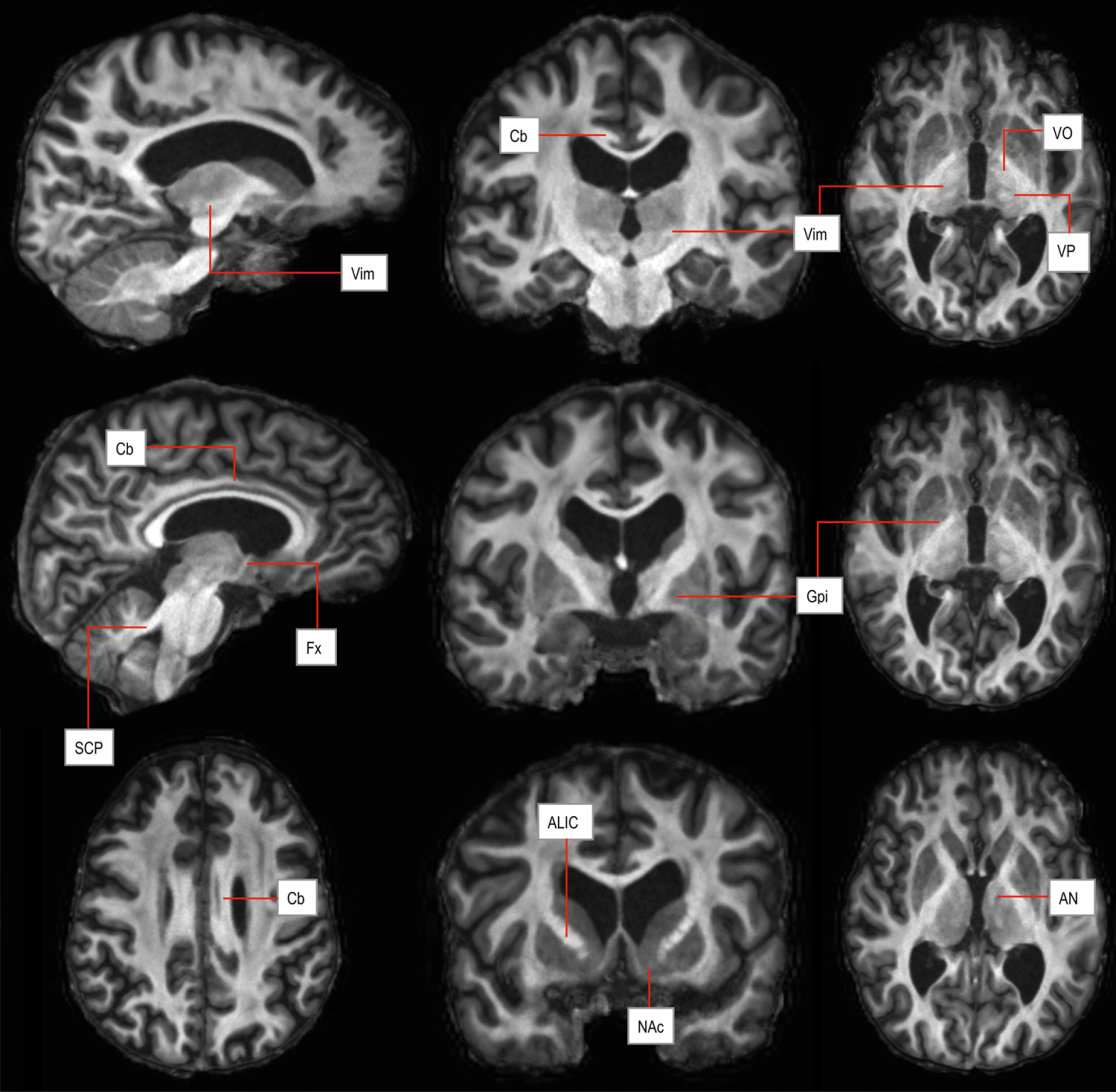
Example of FAT1 weighted imaging, showing detailed thalamic segmentation. ALIC: anterior limb of internal capsule; AN: anterior thalamic nucleus; Cb: cingulum bundle; Fx: fornix; GPi: globus pallidus internus; NAc: nucleus accumbens; SCP: superior cerebellar peduncle; Vim: ventral intermediate nucleus of thalamus; VO: ventrooralis nucleus; VP: ventral posterior thalamic nucleus.

Following a trial-and-error process, the following processing steps yielded the best signal and contrast to noise ratio: The FA maps were registered with the structural T1w image using the first step of the MIST toolbox (Multimodal Segmentation of Subcortical Structures [beta]–FSL v6.0) trained on HCP FA datasets (Visser et al., 2016). The FA map was used with the S0-image (T2 volume) outputted from DTIFIT and the T1 volume. Subsequently, the output from MIST was further processed with fslmaths (FSL v6.0), using the following commands: median filtering (-fmedian), squared root (-sqrt), and multiplication (-mul) with T1 volume. By doing so, the FAT1 images were created (Fig. 2). An additional step to add the extracted skull volume can be carried out, again using fslmaths (-add). Importantly, these images were not used to target the Vim for this cohort but were postoperatively compared with the connectivity-based targeting method, lesion or active DBS stimulation volume, and patient postoperative outcomes (ground truth). The pipeline code is packaged in a docker toolbox which will be made available online. A digital dicom dataset of an example scan is also available to download in Supplementary Digital Data (https://drive.google.com/drive/folders/1WjYj4BPc_pnwrcp7gEEZiHdMN3BXnVSt?usp=drive_link).

**Fig. 3.**
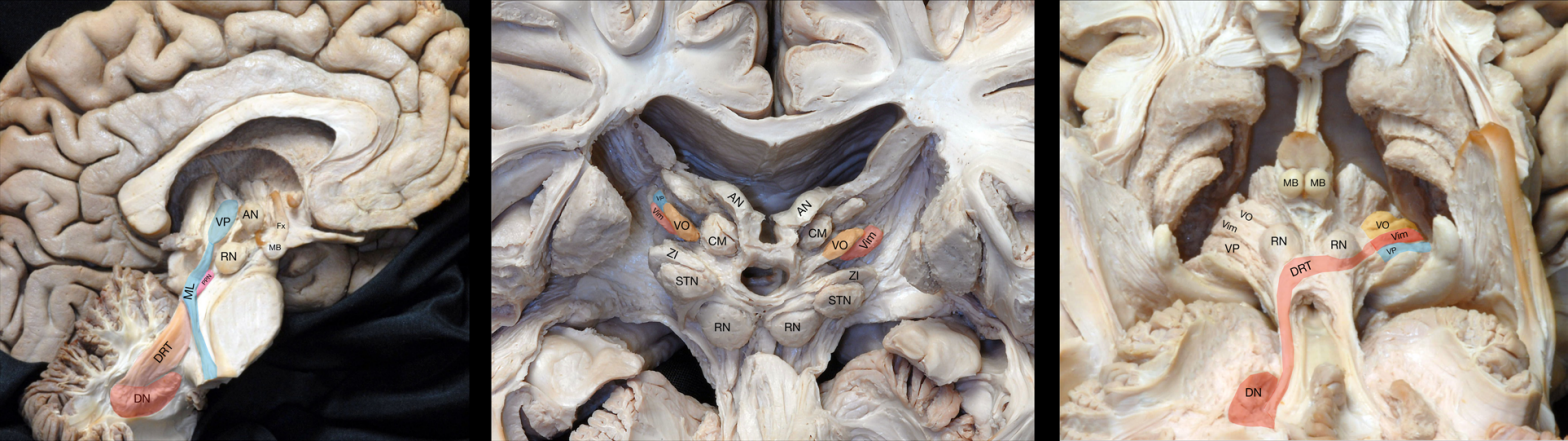
Human brain dissection demonstrating intrathalamic nuclei. Left—Medial white matter dissection of the thalamic VP connecting to the medial lemniscus and of the thalamic AN connecting to the mammillary body. Middle—Anterior view of the thalamic CM, VO, Vim, and VP nuclei. Right—Inferior view of the thalamic VO, VIM, and VP nuclei. DRT is exposed connecting the DN to the RN and VIM. Three formalin-perfused cadaveric human brains were obtained from the anatomical board of the state of Florida. The specimens were frozen at -16^o^C for two weeks before and between the dissections and examined using fibre dissection techniques from medial to lateral, anterior to posterior, and inferior to superior, respectively, to study its thalamic anatomy (Holanda et al., 2020). AN: anterior nucelus; CM: centromedian-parafascicular complex of the thalamus; DN: dentate nucleus; DRT: dentatorubrothalamic tract; Fx: colunm of the fornix; MB: mammillary body; ML: medial lemniscus; PPN: pedunculopontine nucleus; RN: red nucleus; STN: subthalamic nucleus; Vim: ventral intermediate nucleus of the thalamus; VO: ventral oralis nucleus of the thalamus; VP: ventral posterior nucleus of the thalamus; ZI: zona incerta.

To provide an anatomical ground proof, we include a figure from a dissection study carried out on three formalin-perfused cadaveric human brains, obtained from the anatomical board of the state of Florida (USA). The specimens were frozen at -16^o^C for two weeks before and between the dissections and examined using fibre dissection techniques to demonstrate the Vim, VP, and VO (Fig. 3). The technique has been previously discussed in detail (Holanda et al., 2020).

### 2.3 Surgical procedure

#### 2.3.1 Intraoperative MRI acquisition

Stereotactic MRI guidance and verification was used during surgery under local anaesthesia. According to previously described methods, a Leksell frame (model G, Elekta Instrument AB, Stockholm, Sweden) was mounted on the patient’s head and stereotactic pre-implantation MRI scans (T2, Proton Density, and T1-3D MPRAGE) were acquired (Akram et al., 2018;Foltynie et al., 2011;Holl et al., 2010).

#### 2.3.2 Targeting

Intraoperative, stereotactic MRI scans were distortion corrected using the MRI scanner toolbox and then co-registered with the preoperative connectivity segmentation maps to plan two surgical targets: one conventional atlas target using AC-PC coordinates, and a second target using connectivity. Depth was set at Z = 0 for both targets. The atlas coordinates were defined as: X = 12–14 mm and Y = (AC-PC length/3) − 2 mm anterior to PC. The connectivity target was calculated per patient using the thalamic segmented clusters, intending to include as much of the connectivity defined Vim, that is, at the thalamic area with the ipsilateral M1 cluster overlap with the contralateral dentate cluster without encroaching on the ipsilateral S1 cluster (i.e., VP). Since connectivity is influenced by some limitations such as thresholding, the final target was chosen by the surgeon as informed by a combination of the connectivity defined Vim, the atlas coordinates, and intraoperative testing.

#### 2.3.3 Deep brain stimulation (DBS)

A frontal burr hole around the coronal suture was made in line with the planned trajectory. After perforation of the meninges and adequate haemostasis, a 1.5 mm diameter, 2 mm bare tip radiofrequency probe was advanced to the target using dynamic impedance recording. The implantation effect on tremor was assessed in the outstretched contralateral upper limb during probe insertion. The radiofrequency probe was then replaced with the DBS lead, temporarily fixed in situ. Fibrin sealant (Tisseel, Baxter, USA) was used in the burr hole to prevent CSF leak and pneumocephalus (E. A. Petersen et al., 2010). An external stimulator was used to deliver monopolar stimulation to each contact using increasing amplitudes to assess efficacy and side-effect profile. A sign of good placement was considered if a patient experienced transient tingling in the palm upon stimulation. Patients were stressed to elicit the tremor by using verbal recollection and arithmetic tasks. Thresholds for capsular effects and dysesthesia were also assessed. In the case of poor response or unacceptable side-effects, the lead was removed, and the process repeated following appropriate targeting adjustments. Stereotactic MRI was repeated immediately following lead implantation to confirm lead placement. The specific absorption rate (SAR) was kept <0.4 W/kg by reducing the number of acquired T2 slices covering the distal leads to 12–14 (Zrinzo et al., 2011). The leads were then connected to an implantable pulse generator (IPG) implanted in the infra-clavicular region.

#### 2.3.4 Radiofrequency thermocoagulation (RF-T)

In the case of RF-T, the introduction of the radiofrequency probe followed a similar approach as for DBS. Reaching the target, it was examined whether the probe resulted in a stun effect with reduction or disappearance of tremor. The patients were stressed, using verbal recollection and arithmetic tasks, to elicit residual tremor. Stimulation was then performed up to 2 mA at 500us, 133 Hz to check on side effects and to estimate the degree of tremor suppression. In case of poor response or unacceptable side-effects, the lead was removed, and the process repeated following appropriate targeting adjustments. The permanent lesion was then created using 70 °C coagulation for 60 seconds at two or three locations 2 mm apart along one or two adjacent parallel trajectories. Stereotactic MRI was obtained at the end of the radiofrequency coagulation to document lesion location.

### 2.4 Outcome measures

Patients were routinely evaluated by a movement disorders neurologist after surgery. In case of DBS, all DBS contacts were screened once implantation effects had worn off (usually within 2–14 days). Patients were then regularly followed up in clinic to adjust and fine tune stimulation in the first 12 months after surgery. The FTMTRS was used to quantify tremor severity (Stacy et al., 2007). Hand tremor scores (ranged 0–12) were derived from adding the FTMTRS resting (ranged 0–4), postural (ranged 0–4), and intention scores (ranged 0–4) of the treated hand. In cases where FTMTRS assessments were not available, a Clinical Global Impression-improvement (CGI-I) score was measured to estimate outcome in terms of tremor relief of the treated hand (Busner & Targum, 2007;Dec-Ćwiek et al., 2019;Ondo et al., 2001). The CGI-I ranges from 1-7, where 7 is “very much worse” outcome, 4 is no change in outcome, and 1 is “very much improved” outcome. Good response to treatment was defined as either an improvement ≥50% in total FTMTRS, or treated hand tremor scores as compared to the baseline (with ON stimulation in case of DBS). To create one outcome measure without missing data, available FTMTRS improvement scores were translated into a CGI-I score as follows: improvement >75% = CGI-I score 1, improvement 50–75% = CGI-I score 2, improvement 0–50% = CGI-I score 3, no improvement = CGI-I score 4, and worsening (possibly) related to surgery = CGI-I score of 5–7 (depending on the level of severity causing functional impairment). A CGI-I score of ≤2 was considered favourable. Both FTMTRS and CGI-I assessments were performed by experts blinded from imaging data, at least six months after surgery to ensure the stun effect had worn off.

### 2.5 Target analysis

#### 2.5.1 VTA and lesion volume extraction

Immediate postoperative stereotactic MRI (1.5 T Siemens Espree) 1 mm^3^T1 MPRAGE and T2 sequences were imported from DICOM to Nifti. In the case of DBS, Guide XT software (Boston Scientific, Marlborough, Massachusetts, USA) was used to model VTAs around active individual contacts. Optimal settings at the final follow-up point were used. In case of RF-T, thalamic lesions were manually segmented by author [TG] and validated by senior author [HA] using ITK-SNAP (Yushkevich et al., 2006). The VTAs and lesions were then binarised and transformed to the pre-operative structural patient space using FLIRT (6 degrees of freedom, correlation ratio cost function and normal search). To achieve transformed VTA or lesion volumes as close as possible, interpolation voxels were removed using fslmaths (threshold level of 50% by default) (Jenkinson et al., 2012).

#### 2.5.2 Investigating FAT1 based target concordance with the actual target

We carried out a post-hoc analysis of the degree of overlap between the newly created FAT1 based Vim-target, and the VTA or lesion volume achieved with using connectivity targeting (Akram et al., 2018). This was calculated using the DICE coefficient (i.e., two times the overlapping volume divided by the sum of both volumes;Dice, 1945). In order to do so, an FAT1 Vim-target mask was created for each patient by a functional neurosurgical expert [HA] who was blinded of patients’ outcome. First, the FAT1 image was used to target the Vim using Fsleyes (FSL v5.0). Then, a mask centred around the FAT1 Vim-target was created. The radius of this spherical mask (r = 2.5 mm) was based on the average VTA/lesion radius. Finally, the DICE coefficient could be calculated and compared between outcome groups using the created FAT1 Vim-target volume, VTA or lesion volume, and overlapping volume between those two (calculated with fslmaths):

$$\begin{array}{l} DICE=(2\times overlappingvolume/(FAT1targetvolume\\ \;\;\;\;\;\;\;\;\;\;\;\;\;\;\;\;\;\;+VTAorLesionvolume))\times 100 \end{array}$$

Importantly, patients with their VTA at the caudal zona incerta were excluded for the comparison of DICE-scores between outcome groups. We hypothesised that if the FAT1 based Vim-target had a great amount of overlap with the VTA or lesion for patients with a favourable outcome, it would mean that the target was placed within the dentato-thalamo-cortical pathway (and presumably the Vim since the target was set within the thalamus). To further evaluate the predictive value of FAT1 based Vim-targeting on outcome in terms of sensitivity and specificity, the test cut-off value was determined as the point on the receiver operating characteristic (ROC) curve closest to the left-upper corner of the unit square (i.e., the point on the ROC curve closest to a perfect test or 100% sensitivity and specificity) (Habibzadeh et al., 2016).

### 2.6 Statistical analysis

Means with their corresponding standard deviation are presented in continuous variables with a normal distribution (assessed through visual inspection of histograms and Q-Q plots), whereas medians with their corresponding interquartile range (IQR) are presented in continuous variables without a normal distribution. To compare characteristics between patients receiving DBS versus RF-T, and DBS/RF-T outcome groups, univariate statistical analysis was performed. The 2-tailed*t*test (for comparisons of normally distributed continuous variables), Mann–Whitney*U*test (for comparisons of continuous variables without a normal distribution), Fisher’s exact test (for analysis of 2 × 2 tables), and chi-square test (for analysis of*N*× 2 contingency tables) were done when appropriate to identify differences between groups. IBM SPSS Statistics 28.0 (IBM Corporation) was used for calculations.

## 3 Results

### 3.1 Baseline characteristics

Between June 2013 and July 2021, 35 consecutive patients (12 women and 23 men with a mean age of 66 (± 8) years) were treated with DBS (n = 16) or RF-T (n = 19) for severe tremor related to ET (n = 23), or PD (n = 12). On average, patients had a total pre-operative FTMTRS score of 56 (± 22), indicating severe tremor. (Table 1) In total, there were 43 connectivity-guided procedures (including revisional surgery and bilateral procedures). Five patients treated with DBS were explanted before undergoing RF-T and three patients received bilateral DBS. The median follow-up time was longer for patients receiving DBS with 15 months (IQR 12–25), versus RF-T patients with 10 (IQR 6–25) months (Table 1).

**Table 1.** Baseline characteristics of 35 patients undergoing DBS or RF-T for severe medically refractory tremor.

Characteristics	Total (n = 35)	DBS (n = 16)	RF-T (n = 19)	p-value ^a^
Gender, female, n (%)	12 (34)	7 (44)	5 (26)	0.311
Age, mean ± SD	66 ± 8	65 ± 10	66 ± 7	0.839
Diagnosis, n (%)				
ET	23 (66)	9 (56)	14 (74)	0.311
PD	12 (34)	7 (44)	5 (26)	
FTMTRS score pre-op, mean ± SD	56 ± 22	64 (28.5)	51 ± 15.5	0.154
Disease duration (years), median (IQR)	12 (10-48)	19 (8.5-24)	32 (10-57)	0.107
Previous thalamic surgery * , n (%)	7 (20)	0 (0)	7 (37)	**0.009**
Side of operation, n (%)				
L	24 (69)	11 (69)	13 (68)	**0.023**
R	7 (20)	1 (6)	6 (32)	
Bilateral **	4 (11)	4 (25)	0 (0)	
Follow-up time (months), median (IQR)	13 (7-25)	15 (12-25)	10 (6–25)	**0.039**
Favourable outcome, n (%)	25 (71)	13 (81)	12 (63)	0.285
Side effects, n (%)	19 (54)	9 (56)	10 (53)	1.000

a 2-tailed t-test for means, Mann-Whitney U test for medians, Fisher’s exact test for binary variables, and chi-square test for ordinal variables.

* Five patients included in the RF-T column had undergone prior DBS with unfavourable results, and two RF-T patients had undergone RF-T twice, of which only the most recent RF-T cases received diffusion weighted imaging.

** Four patients received bilateral DBS, of which one patient received unilateral stimulation only.

DBS: deep brain stimulation; FTMTRS: Fahn-Tolosa-Marin rating scale; ET: essential tremor; PD: Parkinson’s disease; RF-T: radiofrequency thalamotomy; VTA: volume of tissue activated

### 3.2 Side effects

In 19 (54%) of the 35 patients, one or more (mostly minor) side effects occurred: 15 patients experienced some gait and/or balance problems (all classified minor, of which 6 permanent mild disturbances); 5 patients suffered from minor speech problems (of which 1 DBS patient experienced permanent slight speech slowing with DBS ON); 1 patient reported transient paraesthesia of the treated side after surgery; and 1 patient had a wound infection needing treatment with antibiotics postoperatively. There were 3 (9%) patients with a major side effect: 1 DBS patient suffered from battery lead infection, requiring removal of the entire system; 1 thalamotomy patient reported permanent paraesthesia of the treated side after surgery; and 1 thalamotomy patient reported permanent loss of sense in the lateral side of the treated hand. Side effects did not differ between treatment groups (Table 1).

### 3.3 Outcomes

**Fig. 4.**
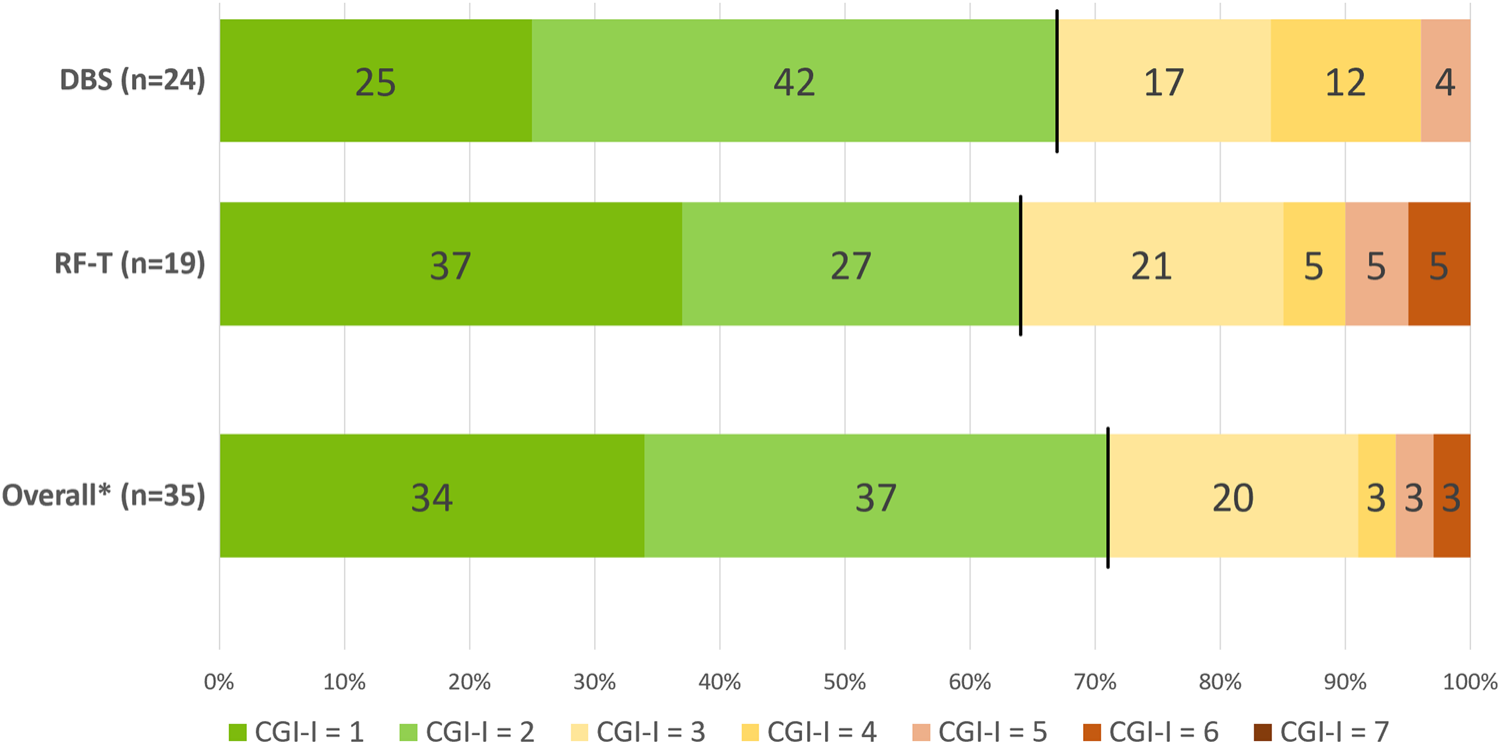
Clinical Global Impression–Improvement (CGI-I) scores for 35 patients undergoing 43 connectivity-guided surgeries: 24 uni/bilateral deep brain stimulations (DBS) and 19 radiofrequency thalamotomies (RF-T).

In total, 25 of 35 (71%) patients receiving DBS and/or RF-T had a favourable outcome at a median follow-up time of 13 (IQR 7–25) months. Of the 17 patients who had treated hand FTMTRS improvement data, the median improvement was 67% (IQR 42–79) and 13 (76%) had an improvement ≥ 50%. Of the ten (29%) patients with an unfavourable outcome, seven (20%) experienced only mild tremor improvement (CGI-I score 3; no meaningful changes in activities of daily living), one (3%) patient had no effect (CGI-I score 4), one (3%) patient experienced some worsening of tremor during the follow-up period (CGI-I score 5), and one (3%) patient experienced worsening of tremor, unilateral hypesthesia, and speech deterioration (CGI-I score 6).Figure 4shows the outcome rates of the 43 surgeries/surgical sides separated by type of treatment, and outcomes of the 35 treated patients according to the CGI-I scale. Baseline characteristics did not differ between outcome groups, both for the total group and separated by type of treatment (Supplementary Table).

### 3.4 FAT1 target analysis

#### 3.4.1 Overlap between FAT1 based vim-target and VTA or lesion

**Fig. 5.**
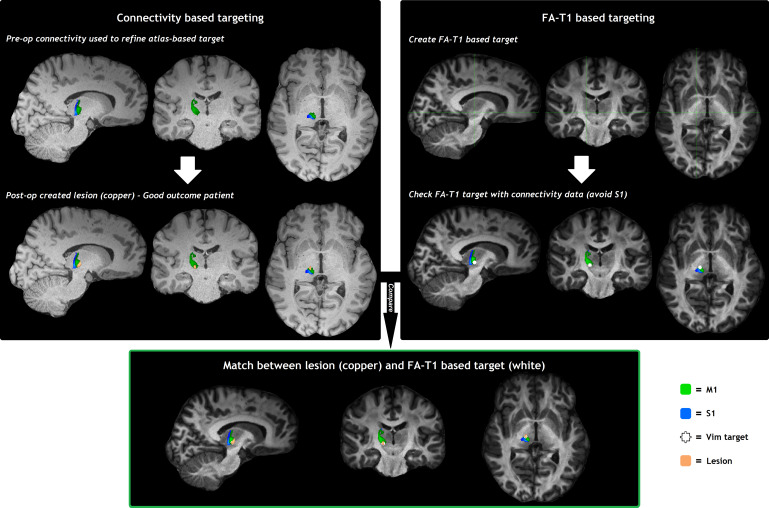
Example of the FAT1 based targeting pipeline and connectivity based surgical pipeline for a good outcome patient showing a great amount of overlap between the FAT1 based Vim target and lesion. FAT1: Fractional anisotropy-T1 weighted imaging; M1: primary motor cortex, S1: primary sensory cortex; Vim: ventral intermediate nucleus of the thalamus

**Fig. 6.**
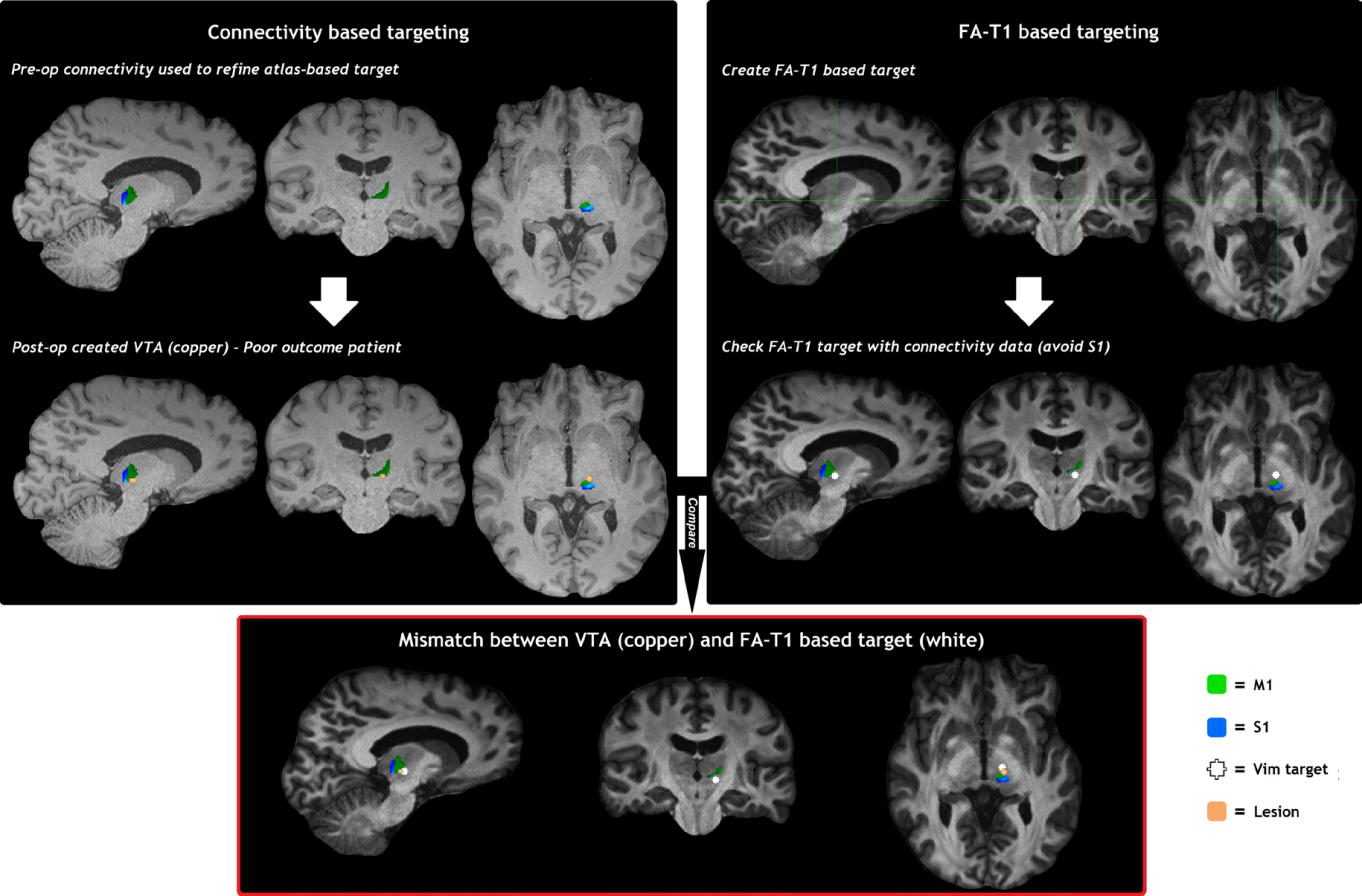
Example of the FAT1 based targeting pipeline and connectivity-based surgical pipeline for a poor outcome patient showing little overlap between the FAT1 based Vim target and VTA. FAT1: fractional anisotropy-T1 weighted imaging; M1: primary motor cortex; S1: primary sensory cortex; Vim: ventral intermediate nucleus of the thalamus; VTA: Volume of Tissue Activated

For 36 of the 43 (84%) surgeries, the preoperatively acquired HARDI and structural imaging were of good quality so that a clear FAT1 image could be created (The poor quality data were mostly caused by motion artefact degrading quality of the diffusion datasets, see subheading 2.2.). For these cases, the neurosurgical expert (HA) was able to distinguish the Vim on the FAT1 images with confidence and create an FAT1 based Vim-target mask (Supplementary Figs. 1 and 2; see also Supplementary Digital Data for an example of FAT1 imaging). There were in total 31 cases eligible for analysis, since five DBS patients had their VTA centred at the caudal zona incerta and were therefore excluded from the analysis as described above. The amount of overlap between the FAT1 based Vim-target and the (connectivity based) VTA or lesion differed significantly between outcome groups: the DICE score was on average 42% (±13) for patients with a favourable outcome, versus 17% (±15) for patients with an unfavourable outcome (MD 25%, 95% CI 14–35, p < 0.0001,Table 2). When analysing DBS and RF-T groups separately, the DICE scores between outcome groups remained significantly different (Table 2).Figures 5and6show the connectivity-based surgical pipeline and the FAT1 based targeting pipeline for a good and a poor outcome patient: in case of a good outcome (Fig. 5), the post-operatively created FAT1 based Vim-target corresponded well with the VTA or lesion. In case of a poor outcome (Fig. 6), however, the FAT1 based Vim-target did not correspond well with the VTA or lesion.

**Table 2.** Overlap in percentage between VTA or lesion and FA-T1 based vim target (i.e., DICE score) in 31 patients receiving vim DBS (n = 16) or RF-T (n = 15) for severe medically refractory tremor.

Characteristics	Total (n = 31) ^§^	Favourable outcome(n = 21)	Unfavourable outcome(n = 10)	p-value ^a^
Mean DICE score ± SD	33.8 ± 17.8	41.7 ± 13.2	17.2 ± 14.9	**<0.0001**
DICE ≥24%, n (%) ^¥^	21 (68)	19 (90)	2 (20)	**<0.0001**
	**DBS (n = 16)**	**11**	**5**	
Mean DICE score ± SD	33.3 ± 18.1	40.5 ± 15.2	17.2 ± 13.9	**0.011**
DICE ≥ 23%, n (%) ^¥^	10 (63)	9 (82)	1 (20)	**0.036**
	**RF-T (n = 15)**	**10**	**5**	
Mean DICE score ± SD	34.3 ± 18.1	42.9 ± 11.2	17.1 ± 17.4	**0.004**
DICE ≥ 26%, n (%) ^¥^	11 (73)	10 (100)	1 (20)	**0.004**

a 2-tailed t-test for means, Mann-Whitney U test for medians, Fisher’s exact test for binary variables.

§ 31 cases were included in the main analysis, since for 12 cases it was either not possible to create an accurate FA-T1 based Vim target (n = 7) AND/OR patients had their VTA targeted at the caudal Zona Incerta (n = 7). (NB All RF-T patients had their lesions initially targeted at the Vim).

¥ Based on the point on the ROC-curve closest to the left-upper corner of the unit square.

DBS: deep brain stimulation; RF-T: radiofrequency thalamotomy; Vim: ventral intermediate nucleus of thalamus; VTA: volume of tissue activated.

#### 3.4.2 FAT1 based targeting

With an area under the ROC-curve of 89%, FAT1 based targeting had an “excellent outcome discrimination” (Hosmer et al., 2013;Power et al., 2013;Supplementary Fig. 3). Based on the point on the ROC-curve closest to the left-upper corner of the unit square, the test cut-off value was determined as a DICE score of 24% for the total group. When applying this cut-off, FAT1 based targeting had a sensitivity of 90%, specificity of 80%, positive predictive value of 90%, and negative predictive value of 80% (Table 2).

## 4 Discussion

This study provides a proof of concept for a new targeting technique combining diffusion with anatomical imaging as a tool to visualise thalamic nuclei, of which the Ventral intermediate nucleus (Vim) of the thalamus can be accurately targeted for patients undergoing thalamic surgery for severe tremor. High-resolution and high-fidelity fractional anisotropy (FA, a product of diffusion tensor imaging) fused with structural imaging realises a visually clear thalamic segmentation. This is not only achievable in a clinically feasible timescale, but also an accurate method to target the Vim.

### 4.1 Defining the sweet spot

In this study, we refer to the tremor target area, as defined by FAT1 as the Vim in keeping with surgical convention, especially that our target is at AC-PC level (i.e., z = 0). This is important to highlight since there are anatomical discrepancies between various classification systems when defining the Vim (Mai & Majtanik, 2019). Furthermore, others have targeted areas inferior and posterior to the Vim with good results in what is described as the caudal zona incerta (cZI), the posterior subthalamic area (PSA), or the prelemniscal radiations (Raprl). It is thought that this is the area where cerebellothalamic fibres spread out and enter the Vim. The target region has been described as a "hypointensity" on the WMnulled/FGATIR sequences (Neudorfer et al., 2022) or as a "rubral wing" (Bot et al., 2023). This has been described in a letter byMiddlebrooks et al. (2022). To keep it simple, and regardless of terminology, we suggest that the target is in the area of the main cerebellar input into the motor thalamus. We target this area inside the thalamus at the AC-PC level.

### 4.2 Limitations of tractography-based segmentation and incorporation of FAT1 based targeting

In our experience, tractography (connectivity)-based segmentation has proven to be useful in targeting the Vim for tremor; however, it has several limitations (Akram et al., 2019). Moreover, we have also shown that there are anatomical bottlenecks that may influence the resulting segmentation, mainly the volume of the superior cerebellar peduncle (Ferreira et al., 2021). The results can also be influenced by the accuracy of the defined ROI seeds, mainly the ventral surface of the thalamus, which, in our experience, is difficult to ascertain using an automated pipeline. This may cause the resulting connectivity-based clusters to be higher along the nucleus (i.e., more superior and anterior) than expected. Another problem is deciding on an arbitrary threshold that would result in accurate representation of the anatomical regions, since this can vary from patient to patient.

What we have found from experience is that the connectivity-defined thalamic cluster that is most useful for targeting is the area connected to the ipsilateral S1 (representing the VP nucleus). This is often used as the posterior limit of the target with an appropriate safety margin (whether this is for DBS or lesion). Being able to dispense of tractography altogether with these limitations and generating an (FAT1) targeting map that incorporates the diffusion signal with the anatomical MR image is a promising step forward.

We highlighted that the Vim can be visualised using FA maps alone. It is feasible that the FA map generated using the pipeline described here (having high contrast-to-noise ratio (CNR), high signal-to-noise ratio (SNR), and being meticulously corrected for distortion) can be used as a standalone map for targeting the Vim. However, FAT1 maps still provide higher resolution, SNR, and CNR by virtue of obtaining higher resolution, and more anatomical detail of grey matter structures from the T1 map. More importantly, most commercially available navigation and targeting software do not handle the fusion between FA and T1 scans well, potentially introducing fusion errors affecting targeting. FAT1 maps can be used for targeting as well as trajectory planning without needing to resort to fusion except with the stereotactic scan which is achieved without difficulty owing to the T1 component. Lastly, there is an opportunity to train machine-learning classifiers on labelled FAT1 structures to segment the same on T1 scans. This is ongoing work that has been showing promise.

Although the work presented in this study is focused on targeting the Vim, the FAT1 image provides good contrast to noise ratio demarcating other thalamic nuclei (the anterior nucleus, the mediodorsal thalamus, the thalamic ventralis oralis complex nucleus, and the centromedian and parafascicularis nuclei) as well as other potential brain targets (e.g., the anterior limb of the internal capsule and the cingulate bundle in surgery for mental disorders; and the pallido-capsular border for targeting the Gpi, etc.;Figs. 2and3). Other potential applications could be in tumour surgery where diffusivity may help better define tumour margins. This is the first iteration of this form of imaging, and it is possible that further refinements would lead to better quality imaging in future. One is able to investigate the potentials of FAT1 imaging by downloading an example uploaded as a Supplementary Digital Data (https://drive.google.com/drive/folders/1WjYj4BPc_pnwrcp7gEEZiHdMN3BXnVSt?usp=drive_link). Furthermore, the FAT1 pipeline code is packaged in a docker toolbox which will be made available online.

### 4.3 Limitations of present study and future directions

This study included severe tremor patients suffering from different types of diseases (e.g., PD and ET). Although no differences in outcome rates or other characteristics between types of diseases were observed in this cohort, one would ideally study a more homogenous patient group. Furthermore, improvement in FTMTRS score for treated side was missing in 18 surgeries, since this was not measured routinely, which justified the use of CGI-I to avoid attrition bias (Busner & Targum, 2007;Dec-Ćwiek et al., 2019;Ondo et al., 2001). While more subjective than the FTMTRS, CGI-I score has been widely used to assess surgical outcome in tremor research (Pahwa et al., 2019;Papapetropolous et al., 2019;Vittal et al., 2017). Additionally, outcome assessments were blinded from imaging data and vice versa, and our outcomes are in line with previous studies (Akbostanci et al., 1999;Bot et al., 2021;Dallapiazza et al., 2019;Jankovic et al., 1995), further strengthening the accuracy of outcome measurement in our work. Eventually, the focus of this work was on the technicality of targeting rather than clinical outcomes. Further work using dedicated tremor rating scale such as the FTMTRS is warranted to further confirm our findings.

In this work, FAT1 maps were generated using HCP style diffusion acquisitions. This may be an overkill, nevertheless, due to advances in multiband scanning; acquisition time (TA) is relatively short (20 minutes for dMRI). Future work will seek to optimise acquisition parameters, improve the final product, as well as reduce scanning time. What is most important in our opinion is acquiring the diffusion datasets in pairs of scans with reverse phase encoding direction to enable post-processing correction of distortion and improvement in SNR.

Since this is the first description of FAT1 based targeting, we chose to compare this method with our gold standard method, including a combination of atlas-defined and connectivity-defined targeting. Therefore, these retrospective results should be interpreted with caution. For example, we did not perform any multivariate testing, correcting the results of this small cohort for other variables such as deviation between the planned target and resulting lesion or DBS contact point. Further, we were unable to create detailed FAT1 images in 16% of the patients due to significant motion artefact and poor quality scans. This mainly occurred in the earlier cohort and we suspect that the expertise we gained over the years with scanning and processing the images has resulted in better FAT1 maps. We will explore this in more detail in future patients. Still, we think it is achievable to create FAT1 weighted images for all patients in the future, owing to better MRI acquisitions nowadays. Our next objective will therefore be to implement this method for future Vim targeting and, to further validate, in prospective research.

FAT1 requires several post-processing steps to generate. This is a limitation. Other, faster MR sequences that do not rely on diffusion MRI, such as fast gray matter acquisition T1 inversion recovery (FGATIR) and proton density, have been shown to be very useful in delineating the Vim. Future work could also be carried to compare these sequences. Having said that, in the last 4 years, we have managed to cut down processing time from around 4 hours to under 60 minutes, simply with improvements in computational power alone. We suspect that with improvements in image processing led by AI, we can generate these maps in a much shorter time, even online immediately after image acquisition. Moreover, using 7T acquisitions to generate FAT1 maps may provide even better CNR to visualise other poorly visualised nuclei and is something to be explored in future.

## 5 Conclusion

FAT1 imaging is a new, high-resolution, and high-fidelity modality that combines diffusion and anatomical MRI. It provides a fast and efficacious way of targeting the ventral intermediate nucleus of the thalamus. In this study, FAT1 based targeting was highly accurate in predicting outcomes after deep brain stimulation and radiofrequency thalamotomy. We submit that this targeting tool may be useful for future patients undergoing thalamic surgery for severe tremor.

## Supplementary Material

Supplementary Material

## Data Availability

The authors confirm that the processed data (e.g., patient characteristics, outcome measurements, and FAT1 targeting data) supporting the findings of this study are available within the manuscript and itsSupplementary Materials. The MRI sequences used for this study are not publicly available due to their containing information that could compromise the privacy of research participants. Still, an FAT1 digital dicom dataset of an example scan (acquired on a 3T Siemens Prisma system using standardised Connectomic / HCP style acquisitions as used in the Aging HCP (Bookheimer et al., 2019)) is available to download: https://drive.google.com/drive/folders/1WjYj4BPc_pnwrcp7gEEZiHdMN3BXnVSt?usp=drive_link Further, a standalone toolbox with the processing pipeline packaged in a docker is available to download and test on request from the corresponding author, HA. The current version is optimised on HCP style data and will be undergoing continuous support and improvements in future releases.
