# Supplementary material for: FAT1 weighted MRI: Diffusion meets anatomical imaging and application in thalamic surgery for tremor
Source: Imaging Neurosci (Camb). 2024 Apr 25;2:imag-2-00139. doi: 10.1162/imag_a_00139 (PMC12247582; doi:10.1162/imag_a_00139)
Supplement: Supplementary Material [file imag_a_00139-supp.pdf]

SUPPLEMENTAL MATERIAL

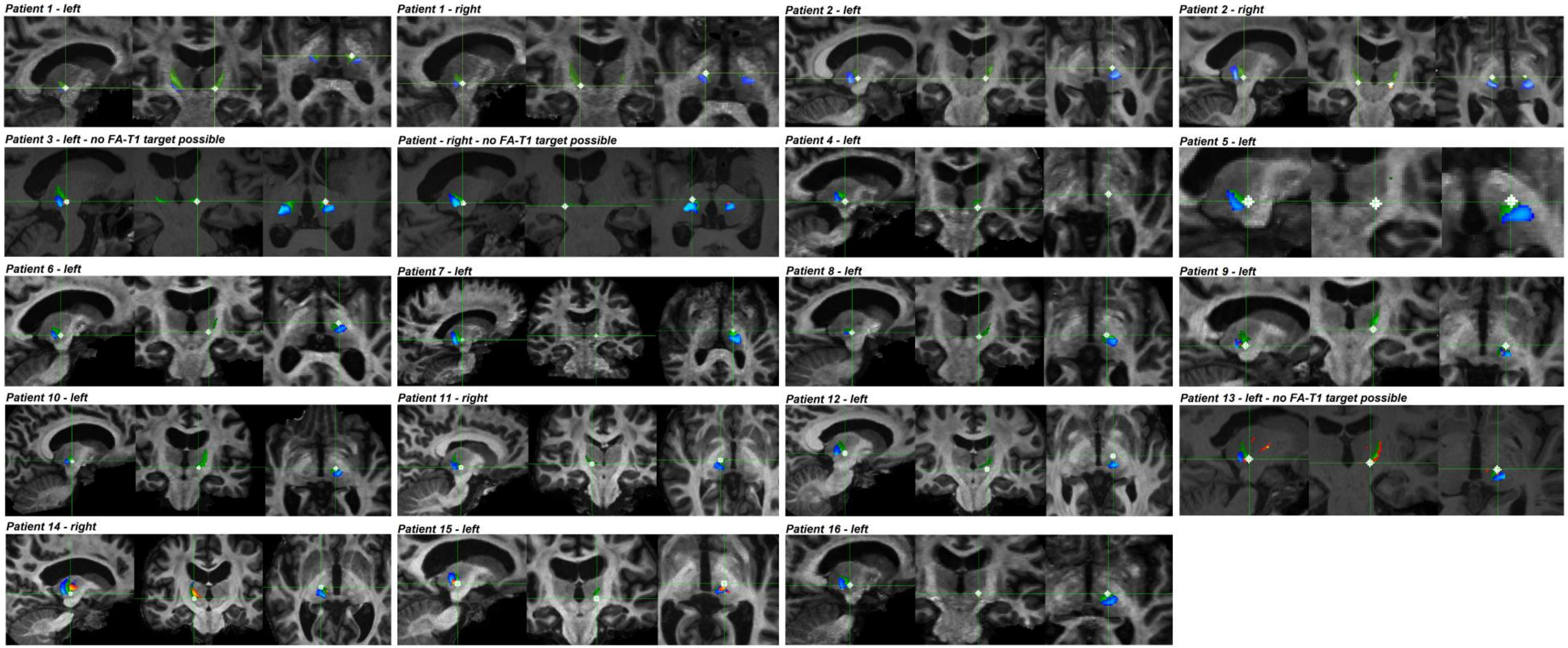

Supplementary Figure 1 FAT1 based Vim targeting for 16 patients who underwent deep brain stimulation

FAT1: fractional anisotropy-T1 weighted imaging; White: Vim-target (NB for patients 3 (bilateral) and 13, FAT1 based targeting was not possible); green: connectivity based thalamic segmentation of ventrolateral nucleus; blue: connectivity based thalamic segmentation of ventroposterior nucleus

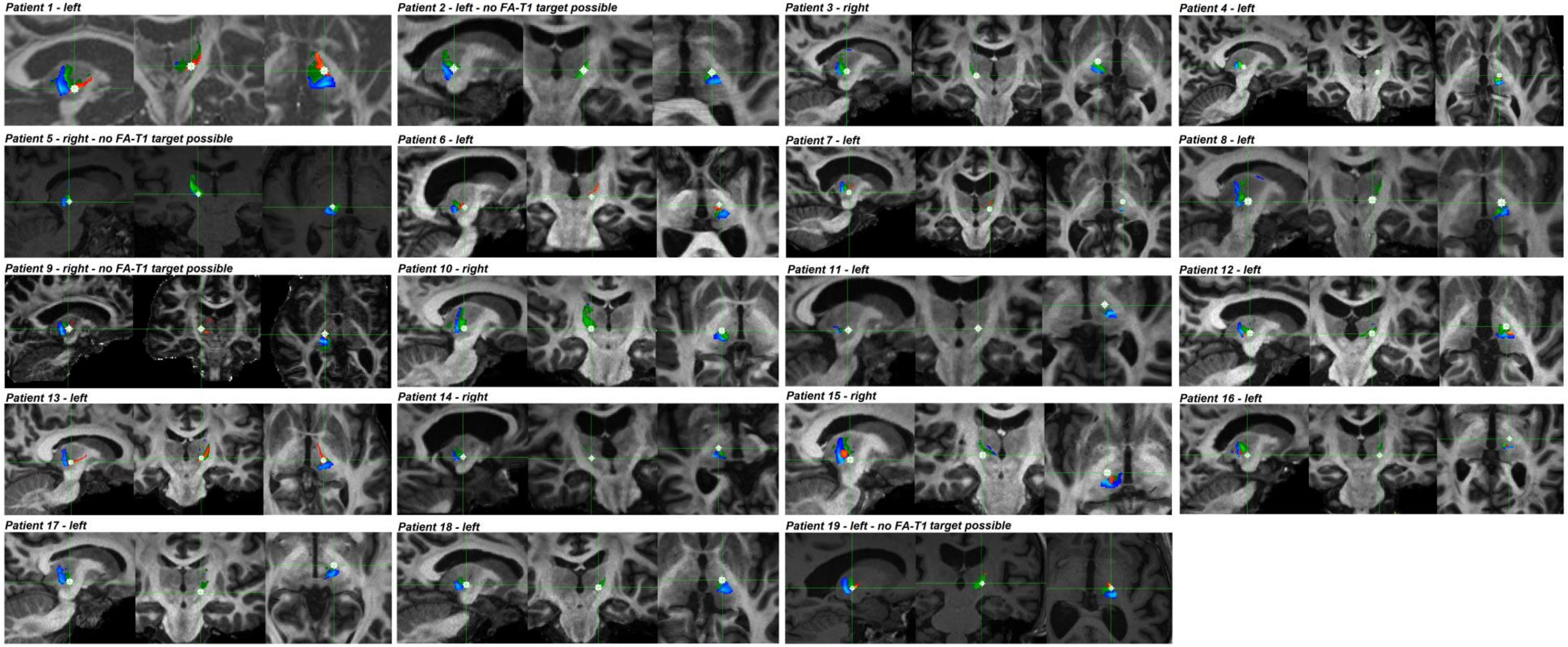

Supplementary Figure 2 FAT1 based Vim targeting for 19 patients who underwent radiofrequency thalamotomy

FAT1: fractional anisotropy-T1 weighted imaging; White: Vim-target (NB for patients 2, 5, 9 and 19, FAT1 based targeting was not possible); green: connectivity based thalamic segmentation of ventrolateral nucleus; blue: connectivity based thalamic segmentation of ventroposterior nucleus

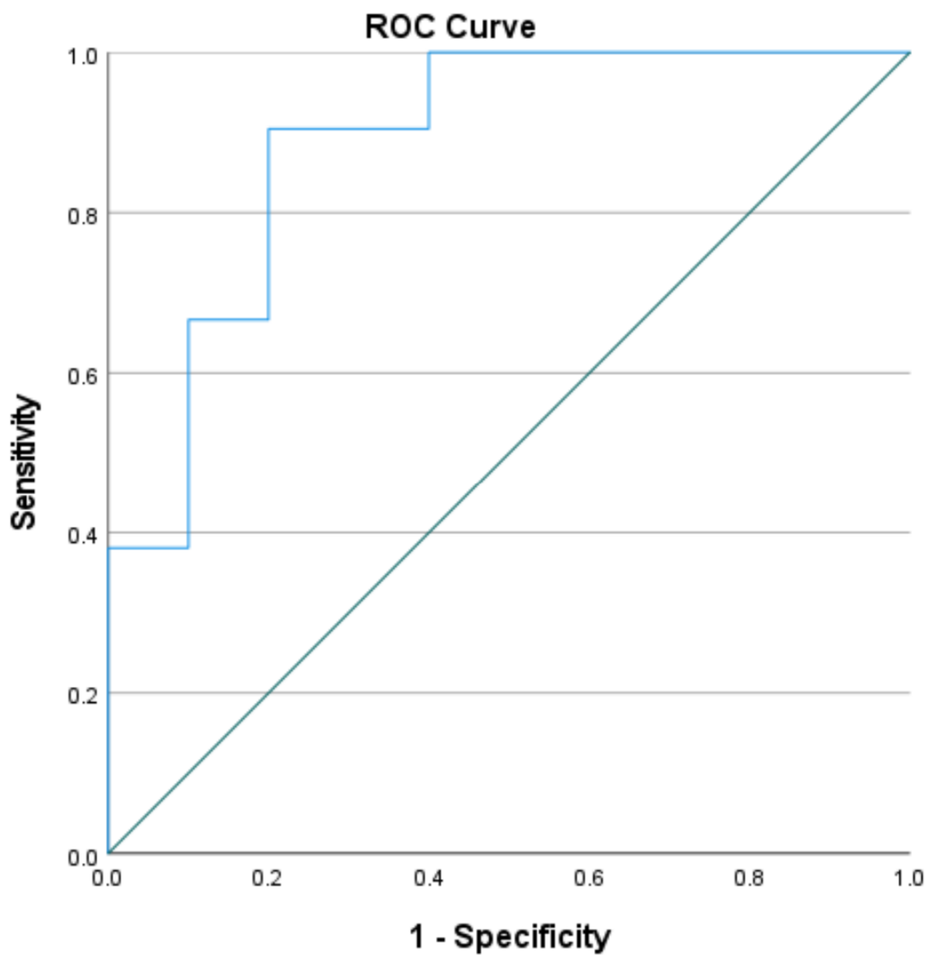

Supplementary Figure 3 Receiver operating characteristic (ROC) curve showing sensitivity and 1-specificity for predicting outcome after thalamic surgery through FAT1 based targeting

Supplementary Table Baseline characteristics of 43 connectivity guided DBS or RF-T surgeries for severe medically refractory tremor, analysed by outcome

Characteristics	Total (n=43)	Favourable (n=28)	unfavourable (n=15)	p-value[a]
**Gender, female, n (%)**	14 (33)	9 (32)	5 (33)	1.000
**Age, mean ± SD**	65.4 ± 8.1	66.9 ± 8.5	62.4 ± 6.6	0.081
**Diagnosis, n (%)**				
ET	27 (63)	16 (57)	11 (73)	0.342
PD	16 (37)	12 (43)	4 (27)	
**Disease duration (years), median (IQR)**	12.0 (10.0-47.0)	12.0 (9.3-46.0)	33.0 (11-50)	0.385
**FTMTRS score pre-op, mean ± SD**	58.1 ± 24.4	59.9 ± 26.3	54.2 ± 19.8	0.506
**Previous thalamic surgery*, n (%)**	7 (16)	4 (14)	3 (20)	0.680
**Left sided surgery**, n (%)**	32 (74)	19 (68)	13 (87)	0.276
**Follow-up time (months), median (IQR)**	14.0 (8.0-25.0)	13.5 (7.0-18.8)	25.0 (10.0– 29.0)	0.250
**Type of treatment**				
**DBS, n (%)**	24 (56)	16 (57)	8 (53)	1.000
**RF-T, n (%)**	19 (44)	12 (43)	7 (47)	
**Side effects, n (%)**	22 (51)	15 (54)	7 (47)	0.755
Present at long term follow-up, n (%)	14 (33)	9 (32)	5 (33)	1.000
**Characteristics**	**DBS (n=24)**	**Favourable (n=16)**	**unfavourable (n=8)**	**p-value[a]**
**Gender, female, n (%)**	9 (38)	6 (38)	3 (38)	1.000
**Age, mean ± SD**	64.6 ± 9.0	66.5 ± 9.5	60.9 ± 7.0	0.154
**Diagnosis, n (%)**				
ET	13 (54)	8 (50)	5 (62)	0.792
PD	11 (46)	8 (50)	3 (38)	
**Disease duration (years), median (IQR)**	11.0 (8.5-44.0)	10.0 (8.5-24.0)	25.0 (7.3-49.3)	0.281
**Previous thalamic surgery, n (%)**	0 (0)			
**Left sided surgery, n (%)**	19 (79)	12 (75)	7 (88)	0.631
**Follow-up time (months), mean ± SD**	18.9 ± 8.7	17.8 ± 7.8	21.3 ± 10.7	0.428
**Side effects, n (%)**	12 (50)	9 (56)	3 (38)	0.667
Present at long term follow-up, n (%)	10 (42)	8 (50)	2 (25)	0.388

Characteristics	RF-T (n=19)	Favourable (n=12)	unfavourable (n=7)	p-value[a]
**Gender, female, n (%)**	5 (26)	3 (25)	2 (29)	1.000
**Age, mean ± SD**	66.3 ± 6.9	67.5 ± 7.2	64.1 ± 6.4	0.321
**Diagnosis, n (%)**				
ET	14 (74)	8 (67)	6 (57)	0.802
PD	5 (26)	4 (33)	1 (14)	
**Disease duration (years), median (IQR)**	33.0 (10.0-57.0)	26.5 (9.3-57.8)	33.0 (11.0-51.0)	1.000
**Previous thalamic surgery, n (%)**	7 (37)	4 (33)	3 (43)	1.000
**Left sided surgery, n (%)**	13 (68)	7 (58)	6 (86)	0.333
**Follow-up time (months), mean ± SD**	13.7 ± 10.0	11.6 ± 10.0	17.3 ± 9.5	0.239
**Side effects, n (%)**	10 (53)	6 (50)	4 (57)	1.000
Present at long term follow-up, n (%)	4 (21)	1 (8)	3 (43)	0.117

DBS: deep brain stimulation; ET: essential tremor; MNI: Montreal Neurological Institute; PD: Parkinson's disease; RF-T: radiofrequency thalamotomy; VTA: volume of tissue activated

a.2-tailed t-test for means, Mann-Whitney U test for medians, Fisher's exact test for binary variables, and chi-square test for ordinal variables

** Five RF-T patients had undergone previous DBS with unfavourable results, and two RF-T patients had undergone RF-T twice, of which only the most recent RF-T cases contain connectivity data and are thus included.*

*** Four patients received bilateral DBS, of which one patient received unilateral stimulation only and thus only included once in the analysis*

SUPPLEMENTARY DIGITAL DATA

FAT1 scan example, acquired on a 3T Siemens Prisma system using standardised Connectomic / HCP style acquisitions as used in the Aging HCP (Bookheimer et al., 2019).

https://drive.google.com/drive/folders/1WjYj4BPc_pnwrcp7gEEZiHdMN3BXnVSt?usp=drive_link

A standalone toolbox with the processing pipeline packaged in a docker is available to download and test on request from the corresponding author, HA. The 1st version is optimised on HCP style data and will be undergoing continuous support and improvements in future releases.
